# Quantification of excretory renal function and urinary protein excretion by determination of body cell mass using bioimpedance analysis

**DOI:** 10.1186/s12882-015-0171-9

**Published:** 2015-10-27

**Authors:** Stefan Flury, Johannes Trachsler, Albin Schwarz, Patrice M. Ambühl

**Affiliations:** Division of Nephrology, Stadtspital Waid, Tièchestrasse 99, 8037 Zürich, Switzerland; Current address: Imperial College Renal and Transplant Centre, Hammersmith Hospital, Du Cane Road, London, W12 0HS UK

**Keywords:** Bioimpedance analysis, Body cell mass, Creatinine clearance, Glomerular filtration rate, 24 h urine collection

## Abstract

**Background:**

Creatinine clearance (CrCl) based on 24 h urine collection is an established method to determine glomerular filtration rate (GFR). However, its measurement is cumbersome and the results are frequently inaccurate. The aim of this study was to develop an alternative method to predict CrCl and urinary protein excretion based on plasma creatinine and the quantification of muscle mass through bioimpedance analysis (BIA).

**Methods:**

In 91 individuals with normal and impaired renal function CrCl was measured from 24 h urine excretion and plasma creatinine concentration. A model to predict 24 h-creatininuria was developed from various measurements assessing muscle mass such as body cell mass (BCM) and fat free mass (FFM) obtained by BIA, skinfold caliper and other techniques (training group, *N* = 60). Multivariate regression analysis was performed to predict 24 h-creatininuria and to calculate CrCl. A validation group (*N* = 31) served to compare predicted and measured CrCl.

**Results:**

Overall (accuracy, bias, precision, correlation) the new BIA based prediction model performed substantially better compared with measured CrCl (P_15_ = 87 %, bias = 0, IQR of differences = 7.9 mL/min/1.73 m^2^, *R* = 0.972) versus established estimation formulas such as the 4vMDRD (P_15_ = 26 %, bias = -8.3 mL/min/1.73 m^2^, IQR = 13.7 mL/min/1.73 m^2^, *R* = 0.935), CKD-EPI (P_15_ = 29 %, bias = -7.0 mL/min/1.73 m^2^, IQR = 12.1 mL/min/1.73 m^2^, *R* = 0.932, Cockcroft-Gault equations (P_15_ = 55 %, bias = -4.4 mL/min/1.73 m^2^, IQR = 9.0 mL/min/1.73 m^2^, *R* = 0.920). The superiority of the new method over established prediction formulas was most obvious in a subgroup of individuals with BMI > 30 kg/m^2^ and in a subgroup with CrCl > 60 mL/min/1.73 m^2^. Moreover, 24 h urinary protein excretion could be estimated accurately by normalization with 24 h-creatininuria derived from BIA based BCM.

**Conclusion:**

Prediction of CrCl based on estimated urinary creatinine excretion determined from measurement of BCM by BIA technique is both accurate and convenient to quantify renal function in normal and diseased states. This new method may become particularly helpful for the evaluation of patients with borderline renal insufficiency and/or with abnormal body composition.

## Background

Glomerular filtration rate (GFR), as a measure of excretory kidney function is an important parameter to make diagnostic or therapeutic decisions as well as a tool to monitor the course of kidney disease. The gold standards to measure GFR are inulin or radioisotope clearance techniques. GFR, however, can also be determined fairly accurate and non-invasive by measuring creatinine clearance, which requires a 24 h urine collection. This method, however, has been replaced widely in clinical practice by estimating GFR using established formulas due to simplicity and ease of use [1, 2].

For GFR estimation in adult patients the validated CKD-EPI and the four-variables-MDRD (4vMDRD) formulas are used most often, whereas the Cockcroft-Gault formula can be applied to estimate creatinine clearance (CrCl). For a range of GFR around and beyond 60 mL/min/1.73 m^2^ the 4vMDRD-formula is less accurate than the CKD-EPI formula. The Cockcroft-Gault formula on the other hand is less precise in predicting renal function in cases of advanced kidney failure. Moreover, this formula overestimates GFR slightly because it determines CrCl [3–8].

All the prediction equations share the common feature to be based on plasma creatinine (PCr), whose production depends mainly on muscle mass, which is not incorporated in the formulas mentioned before. Therefore, in patients with very high or low muscle mass, the estimations are often imprecise [9]. Besides, the formulas are not validated for all situations of clinical practice. For example, the CKD-EPI formula is not validated for adolescents below the age of 18 years or for pregnant women, whereas the 4vMDRD-formula is not validated for the very old (beyond 85 years). Both formulas were developed from cohorts consisting of mainly Caucasians or Blacks with uncertain accuracy in other ethnical groups [10–14]. Moreover, in normal to mild impairment of kidney function, the estimations are highly scattered compared to gold standard methods [6, 15, 16]. Thus, in many situations, it is still more reliable to measure CrCl in order to accurately assess a patient’s kidney function than to rely only on the estimation formulas.

An alternative approach to renal function determination by either 24 h urine collection, radioisotope methods or estimation formulas could be the incorporation of measured muscle mass into the calculation of CrCl. Several methods to analyze tissue distribution are available. Body composition can be determined according to its compartments of fat mass and lean body mass by radiological techniques of dual x-ray absorptiometry (DXA) or bioimpedance analysis (BIA) [17, 18]. An older method relies on prediction of body fat mass and fat free mass by measuring subcutaneous skin folds in a standardized manner [19, 20].

The aim of our study was to elaborate and validate a method based on parameters obtained by BIA to predict CrCl as a measure of excretory renal function without timed urine collection and on the basis of previously published studies by Donadio et al. [21–23]. Additionally, we wanted to evaluate whether other methods of muscle mass determination – i.e. skin fold based measurements, mid arm muscle circumference (MAMC) or other anthropometric parameters such as BMI or waist-hip ratio – may be used alternatively or complementary for predictive models. Furthermore, the developed approach to determine creatinine excretion could be used to estimate 24 h urinary protein excretion, which is a cornerstone to diagnose, classify and monitor proteinuric nephropathies.

## Methods

### Study population

A total of 91 individuals (85 patients consecutively presenting in our outpatient clinic and 6 healthy volunteers) were included in this study. The cohort was divided into a ‘training group’, formed by the first 60 individuals including the 6 healthy volunteers (42 male, 18 female), and a ‘validation group’ (15 male, 16 female). All participants were required to have a stable kidney function, as could be verified by checking plasma creatinine based on local medical records on file or from referral documents. The nephrological diagnoses or reasons for referral consisted of: Chronic kidney disease caused by vascular nephropathy (*N* = 24), diabetic nephropathy (10), glomerulonephritis (13), polycystic kidney disease (7) and various causes (18), patients with kidney transplants (3), nephrolithiasis workup (10), and six healthy volunteers. All laboratory measurements were conducted as part of the diagnostic routine. Similarly, determination of body composition is an integral component of the workup of patients with kidney disease at our institution.

### Anthropometry, lab analyses

In all participants, CrCl was determined based on a single 24 h urine collection and plasma creatinine measurement. The absolute value for measured CrCl (24hU-CrCl) was normalized for estimated body surface area (according to DuBois formula). All individuals received detailed instructions on correct 24 h urine sampling. On the day of consultation, the following anthropometric measurements were performed: Weight, height, waist, hip circumference and upper arm circumference; standardized skin folds were measured with a commercially available caliper instrument. Lean body mass (LBM) was calculated using the skin fold measurements according to the equation of Durnin and Womersley [19]. Midarm muscle circumference (MAMC) was determined using the formula MAMC [cm] = MAC [cm] - 0.314 x TSF [mm]. Finally, waist/hip-ratio was calculated [24, 25]. At the same time, a body impedance analysis using the single frequency body impedance device “BIA 101” (Akern®) was conducted according to manufacturer’s instructions [26–28]. This device delivers impulses and alternating current of 0.8 mA with a frequency of 50 kHz. To measure the resistance (R) and reactance (Xc), two electrodes have to be placed on each extremity of one body half of the patient in supine position. Fat free mass (FFM) and body cell mass (BCM) were obtained from input of the bioimpedance data, body weight and height as well as gender by a specific software (Bodygram 3.0®). Additionally, FFM was also determined by a commercially available handheld BIA technology device (Omron HBF 306 Body Logic Pro Body Fat Analyzer®).

After venipuncture and blood centrifugation plasma creatinine (PCr) was determined in the local laboratory according to the Jaffé method (CREJ2®, Roche Diagnostics). In each individual’s collection urine creatinine was determined and creatinine clearances were calculated. In addition, in 36 out of the 91 participants the total amount of protein was determined and protein and creatinine were measured from a spot urine sample. For both quantifications the same turbidimetric method was used (TPUC3®, Roche Diagnostics) on a Cobas®, Roche Diagnostics, platform.

### Statistical analyses, model development

The prediction rule for estimating creatinine clearance based on anthropometric data was derived in three steps. First, a multivariate regression model was developed to predict 24 h-creatininuria (24hU-Cr) obtained from urine collection. Second, 24 h-creatininuria determined by this regression model was used to calculate the model based creatinine clearance (mCrCl) according to the equation mCrCl = m24hU-Cr [mmol]/PCr [μmol/L]/1440 [min]. After adjusting for the different dimensions of the enumerator (mmol) and the denominator (μmol), the result in L/min was multiplied by factor 1000, to receive the usual clearance unit mL/min (analogously to the calculation of CrCl from 24 h urine collection). For ease of comparison, absolute values of mCrCl were normalized for body surface area (BSA) (according to the DuBois formula). Finally, the prediction model was evaluated in a "validation group”. In order to assess the performance of each method to estimate GFR or CrCl, correlation analyses and a modified Bland-Altman plot between measured CrCl from timed urine collection (24hU-CrCl) and the various GFR and CrCl estimation formulas (4vMDRD, CKD-EPI, Cockcroft-Gault formula), as well as the CrCl calculated from the BIA-BCM based model 24 h-creatininuria (mCrCl) were applied [29]. Besides, the following three parameters were used to compare between the mentioned estimation methods and 24hU-CrCl: bias (median difference between GFR resp. CrCl estimate and 24hU-CrCl) and absolute bias (median difference between │GFR resp. CrCl estimate − 24hU-CrCl│); precision (IQR and P25, P75 of differences between GFR resp. CrCl estimate and 24hU-CrCl); accuracy (absolute number and percentage of the estimates that were within 15 % range (P_15_) and/or 30 % range of 24hU-CrCl (P_30_)). The Wilcoxon test and the McNemar test were used, where applicable, to compare the estimation methods with measured CrCl regarding stastically significant differences of GFR results and accuracy values, respectively.

In addition, for the 36 patients in whom the amount of urinary proteins were quantified, we calculated 24 h urinary protein excretion by extrapolation of the single spot specimen urinary protein/creatinine ratio (UPCR) using the new model based 24 h-creatininuria according to pPU = m24hU-Cr x UPCR. The extrapolated values of urinary protein excretion and the protein/creatinine ratios (with and without correction for body surface) were compared to the amounts obtained from 24 h protein excretion by correlation analysis and modified Bland-Altman-analysis. Additionally, bias (median difference between pPU and measured 24hU-PU), precision (IQR and P25, P75 of differences between pPU method and measured 24hU-PU), accuracy (absolute number and percentage of pPU values that lied within range 15 % (P_15_), and/or 30 % range of measured 24hU-PU (P_30_) were calculated.

All measurements in patients were performed as part of routine examinations. Healthy volunteers gave verbal informed consent on file to participate in the measurements. The study protocol is in accordance with and was formally approved by the institutional review board (ethics committee of the Canton of Zurich).

All analyses were conducted using the Statistical Package for the Social Sciences (SPSS 19.0, SPSS, Chicago, Ill., USA), Medcalc for Windows (Medcalc Version 13.0, MedCalc Software, Ostend, Belgium) was used for the figures.

## Results

Patient characteristics are listed in Tables 1 and 2 for the training and validation group respectively. In the training group significant correlations with a Pearson’s correlation coefficient (R) beyond 0.5 with 24-h urine creatinine (24hU-Cr) excretion were detected for the following parameters: FFM and BCM detected by whole body bioimpedance analysis, FFM obtained by Omron® BIA handheld device as well as for body weight, body height, estimated body surface area and lean body mass calculated from skin fold measurements. The correlation coefficients of each recorded parameter with 24hU-Cr are shown in Table 3. The best correlation with *R* = 0.896 was observed with BCM measured by whole body bioimpedance analysis using “BIA 101” (Akern®).

**Table 1:** Patient characteristics training group (*N* = 60)

	Mean ± SD	Median (P25, P75)	Minimum, Maximum
Age; y	53 ± 16	56 (38, 64)	23, 83
Plasma creatinine; μmol/L	196.0 ± 157.0	137 (93.0, 258.0)	60.0, 831.0
24hU Creatinine; mmol	13.68 ± 4.22	13.6 (10.9, 16.4)	5.46, 25.10
24hU-CrCl; mL/min/1.73 m^2^	61.7 ± 34.9	55.0 (28.7, 90.3)	11.5, 136.0
eCrCl (Cockcroft-Gault); mL/min/1.73 m^2^	58.6 ± 37.2	52.4 (27.6, 78.6)	10.5, 175.7
eGFR (4vMDRD); mL/min/1.73 m^2^	47.6 ± 28.9	43.0 (22.8, 70.5)	6.0, 124.0
eGFR (CKD-EPI); mL/min/1.73 m^2^	51.1 ± 32.0	44.0 (23.3, 74.0)	6.0, 120.0
Body weight; kg	83.6 ± 21.2	78.7 (70.0, 94.3)	49.3, 154.6
Body height; cm	173 ± 9	174.5 (167.0, 180.3)	154, 192
BMI kg/m^2^	28.1 ± 6.8	25.8 (23.1, 31.2)	18.6, 56.8
Body Surface Area; m^2^	1.99 ± 0.27	1.97 (1.83, 2.13)	1.49, 2.66
BIA Free Fat Mass; kg	57.8 ± 12.6	57.1 (48.5, 67.2)	31.6, 93.1
BIA Body Cell Mass; kg	29.3 ± 8.2	28.3 (23.1, 34.7)	11.6, 52.5
Abdominal circumference; cm	101 ± 18	98 (89, 113)	72, 146
Hip circumference; cm	103 ± 13	102 (95, 110)	80, 147
Waist/hip ratio	0.98 ± 0.09	0.98 (0.93, 1.03)	0.75, 1.13
MAMC; cm	27.8 ± 4.0	27.7 (25.1, 30.2)	21.5, 40.5
LBM (skinfolds); kg	56.9 ± 11.7	57.4 (48.7, 65.3)	35.0, 88.2

**Table 2:** Patient characteristics validation group (*N* = 31)

	Mean ± SD	Median (P25, P75)	Minimum, Maximum
Age; y	57 ± 16	57 (47, 69)	21, 82
Plasma creatinine; μmol/L	174.6 ± 100.1	157 (87.5, 222.5)	68.0, 373.0
Creatinine, 24hU; mmol	11.30 ± 3.42	11.3 (8.73, 14.50)	6.54, 17.80
CrCl; mL/min/1.73 m^2^	58.4 ± 37.5	44.0 (26.7, 89.8)	14.2, 137.0
eCrCl (Cockcroft-Gault); mL/min/1.73 m^2^	54.6 ± 35.2	37.9 (36.4, 86.6)	11.1, 201.1
eGFR (4vMDRD); mL/min/1.73 m^2^	49.0 ± 32.3	34.0 (20.5, 78.5)	10.0, 115.0
eGFR (CKD-EPI); mL/min/1.73 m^2^	52.0 ± 36.2	37.0 (20.5, 84.0)	10.0, 128.0
Body weight; kg	80.5 ± 13.7	76.4 (71.7, 85.3)	51.4, 113.4
Body height; cm	167 ± 10	167 (160, 173)	147, 187
BMI kg/m^2^	28.70 ± 5.3	27.5 (25.2, 29.8)	20.1, 45.4
Body Surface Area; m^2^	1.81 ± 0.61	1.88 (1.73, 2.01)	1.51, 2.25
BIA Free Fat Mass; kg	54.0 ± 11.3	50.0 (44.3, 57.0)	36.9, 91.2
BIA Body Cell Mass; kg	25.2 ± 6.3	24.4 (19.1, 30.1)	14.2, 37.7
Abdominal circumference; cm	104 ± 11	103 (94, 108)	91, 115
Hip circumference; cm	99 ± 13	100 (95, 104)	68, 112
Waist/hip ratio	0.99 ± 0.12	0.98 (0.95, 1.02)	0.74, 1.13

**Table 3:** Bivariate correlation analysis between 24 h-creatininuria and various variables

		Age	PCr	BIA-FFM	BIA-BCM	BIA BCM Omron	Body weight	Body size	BMI	Body surface area	Waist/Hip ratio	LBM skin folds	MAMC
24hU-Cr	R (Pearson)	−0.374	0.195	0.794	0.896	0.780	0.561	0.631	0.323	0.647	0.317	0.696	0.483
	P (2-sided)	0.003	0.136	0.000	0.000	0.000	0.000	0.000	0.012	0.000	0.015	0.000	0.000

The set of all measured parameters mentioned above having a correlation coefficient > 0.5 (see Table 3) was then entered into a stepwise multivariate linear regression model, in order to predict 24 h-creatininuria (m24hU-Cr). In addition, the dichotomous variable “gender” was included into the analysis (“male” = 2, “female” = 1). The best model to predict 24-h urinary creatinine excretion consisted of only two variables, namely body cell mass determined by BIA and gender. This model had a coefficient of determination (R^2^) of 0.81.

The model resulting from the multivariate linear regression analysis is expressed by the following formula:

$$ \mathrm{m}24\mathrm{h}\mathrm{U}-\mathrm{C}\mathrm{r}\left[\mathrm{mmol}\right] = 2.1 + 0.43 \times \mathrm{B}\mathrm{I}\mathrm{A}-\mathrm{B}\mathrm{C}\mathrm{M}\ \left[\mathrm{kg}\right]-0.92 \times \mathrm{Gender}\ \left[\mathrm{f} = 1;\mathrm{m} = 2\right];R = 0.899,\mathrm{SEE} = 1.9 $$

Finally, to obtain an estimation of excretory kidney function, the model based 24 h-creatininuria (m24hU-Cr) was entered into the equation for creatinine clearance (mCrCl = m24hU-Cr/PCr/1440 min).

**Fig. 1:**
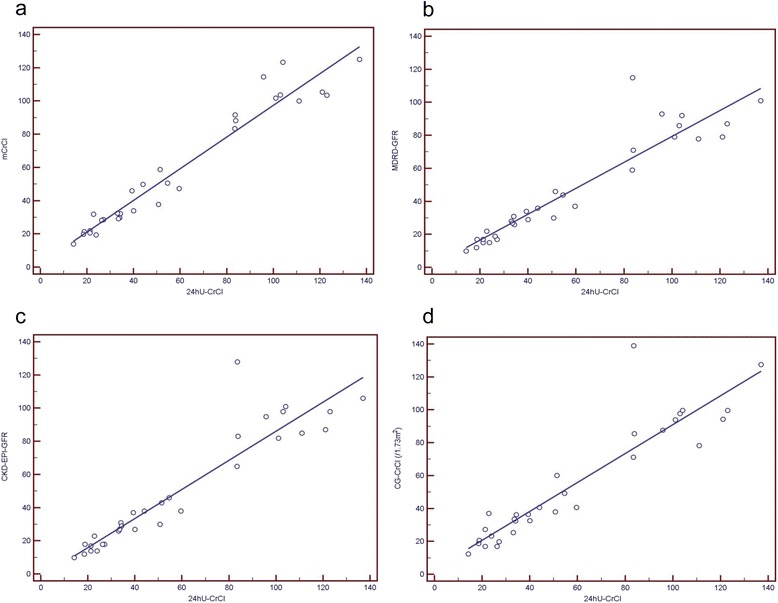
Correlation plots of GFR prediction by different methods with 24hU-CrCl (units of x- and y-axis mL/min/1.73 m^2^). **a** mCrCl: y = 0.95x + 2.19, SEE = 8.83; R = 0.97, *p* < 0.001. **b** 4vMDRD-GFR: y = 0.79x + 0.84, SEE = 11.37; R = 0.93, *p* < 0.001. **c** CKD-EPI-GFR: y = 0.88x -1.53, SEE = 12.99; R = 0.93, *p* < 0.001. **d** CG-CrCl: y = 0.88x +3.26, SEE = 14.24; *R* = 0.92, *p* < 0.001

**Fig. 2:**
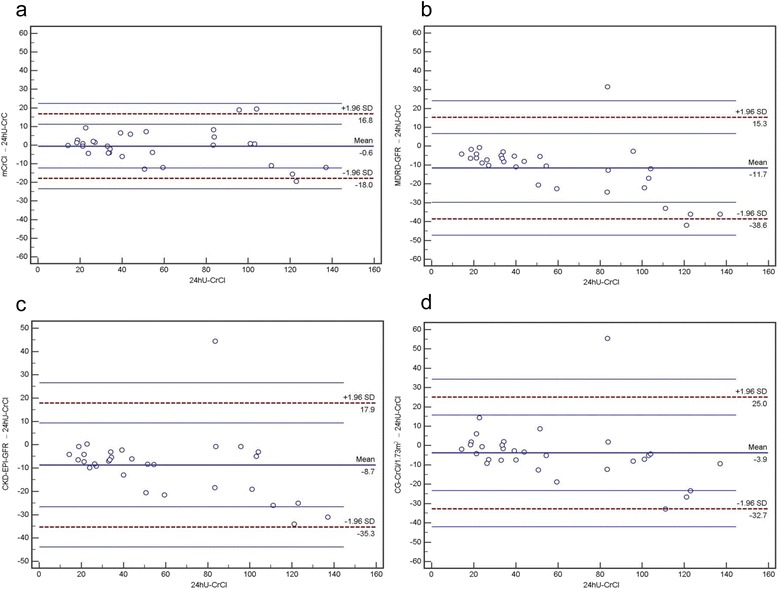
Bland-Altman-Plots of GFR-prediction by different methods in comparison with 24hU-CrCl (units of x- and y-axis mL/min/1.73 m^2^). **a** mCrCl: bias (mean difference) = -0.6, limits of agreement (LoA) = -18.0, 16.8 (dashed lines), 95 % confidence interval of upper and lower LoA (thin lines). **b** 4vMDRD-GFR: bias = -11.7, LoA = -38.6, 15.3. **c** CKD-EPI-GFR: bias = -8.7, LoA = -35.3, 17.9. **d** CG-CrCl: bias = -3.9, LoA = -32.7, 25.0

In the training group, this model performed quite well to predict CrCl, having a correlation with measured CrCl of *R* = 0.970 (*p* < 0.001), SEE = 8.7 mL/min/1.73 m^2^, where the median difference was 7.4 mL/min/1.73 m^2^, and IQR 16.4 mL/min/1.73 m^2^ (see Table 4). In the validation group, the model derived from the training group still correlated strongly with measured CrCl (*R* = 0.972, *p* < 0.001, SEE = 8.8 mL/min/1.73 m^2^; Table 5, Fig. 1). In contrast, GFR values obtained from the 4vMDRD and CKD-EPI formulas corresponded clearly less with measured CrCl (*R* = 0.935, *p* < 0.001, SEE = 11.4 mL/min/1.73 m^2^, and *R* = 0.932, *p* < 0.001, SEE = 13.0 mL/min/1.73 m^2^, respectively). Similarly, correlation of the Cockcroft-Gault equation was even lower for 24hU-CrCl (*R* = 0.920, *p* < 0.001, SEE = 14.2 mL/min/1.73 m^2^). The median difference between measured CrCl and the prediction method of excretory kidney function was lowest for the BCM based model mCrCl (bias = 0, absolute bias = 4.4, IQR = 7.9 mL/min/1.73 m^2^). In contrast, bias, absolute bias and precision for 4vMDRD, CKD-EPI and Cockcroft-Gault (CG) were clearly worse with -8.3, 8.9, IQR = 13.7 mL/min/1.73 m^2^ (median fractional prediction error of 21.8 %); -7.0, 7.2, IQR = 12.1, 7.2 mL/min/1.73 m^2^ (19.8 %); and -4.4, 7.1, IQR = 9.0, 7.1 mL/min/1.73 m^2^ (7.0 %), respectively (Table 5, Fig. 2). Statistically significant differences for eGFR between 4vMDRD and CKD-EPI vs. 24hU-CrCl (*p* < 0.001) and CG-CrCl vs. 24hU-CrCl (*p* = 0.01), but not between the new BCM derived model and 24hU-CrCl (*p* = 0.86), were shown. Regarding accuracy, the BCM derived model showed a significantly better performance in the most important category P_15_. The results of correlation, bias, precision and accuracy considering different subgroups within the validation group according to gender, mCrCl or BMI are shown in Table 5. The better performance of the BCM based model over the other prediction methods is most obvious for 24hU-CrCl > 60 mL/min/1.73 m^2^ and for BMI > 30 kg/m^2^ (involving 4 individuals with a BMI ≤ 34, 2 with a BMI of 35 and 2 with a BMI > 35 kg/m^2^, the maximum being 45.4 kg/m^2^), both with regard to correlation and to accuracy, but also to precision as an indicator of dispersion of the prediction methods (see Table 5).

**Table 4:** Training group: Correlations, bias, precision and accuracy of the different GFR prediction methods and measured 24hU-CrCl

		Correlation coefficient R	SEE^†^	BIAS Median difference^†^	ABSOLUTE BIAS^†^	PRECISION IQR (P25,P75) of differences^†^	ACCURACY	
							P15 (%)	P30 (%)
All subjects (*N* = 60)	mCrCl	0.970***	8.7	7.4	8.5	16.4 (1.1, 17.5)	48 (80)^a^	56 (93)^bcd^
	4vMDRD	0.930***	13.1	−11.7	12.10	13.6 (-19.6, -5.9)	15 (25)	37 (62)
	CKD-EPI	0.950***	10.7	−10.2	10.4	10.6 (-15.5, -4.9)	21 (35)	45 (75)
	CG-CrCl	0.840***	19.3	−4.4	9.2	14.2 (-11.2, 3.0)	26 (43)	51 (85)

***p < 0.001

^†^displayed in mL/min/1.73 m^2^;

^a^ *p* < 0.001 vs the three other methods; ^b^ *p* = 0.001 vs 4vMDRD; ^c^ *p* < 0.05 vs CKD-EPI; ^d^ *p* = 0.18 vs CG

**Table 5:** Validation group: Correlations, bias, precision and accuracy of the different GFR prediction methods and measured 24hU-CrCl

		Correlation coefficient R	SEE^†^	BIAS Median difference^†^	ABSOLUTE BIAS^†^	PRECISION IQR (P25, P75) of differences^†^	ACCURACY	
							P15 (%)	P30 (%)
All subjects (*N* = 31)	mCrCl	0.972***	8.8	0	4.4	7.9 (-4.3, 3.6)	27 (87)^a^	30 (97)^bcd^
	4vMDRD	0.935***	11.4	−8.3	8.9	13.7 (-18.8, -5.2)	8 (26)	25 (81)
	CKD-EPI	0.932***	13.0	−7.0	7.2	12.1 (-15.7, -3.6)	9 (29)	26 (84)
	CG-CrCl	0.920***	14.2	−4.4	7.1	9.0 (-8.7, 0.4)	17 (55)	28 (90)
Male (*N* = 15)	mCrCl	0.958***	11.3	0.8	7.3	15.5 (-11.5, 4.0)	13 (87)	15 (100)
	4vMDRD	0.961***	9.0	−17.0	17.0	21.9 (-28.7, -6.8)	3 (20)	12 (80)
	CKD-EPI	0.967***	9.2	−8.4	8.4	17.1 (- 23.3, -6.2)	4 (27)	14 (93)
	CG-CrCl	0.965***	9.6	−8.8	8.8	11.2 (-15.5, -4.3)	8 (53)	14 (93)
Female (*N* = 16)	mCrCl	0.967***	5.9	−0.6	4.2	7.3 (-4.2, 3.1)	15 (94)	15 (94)
	4vMDRD	0.915***	10.8	−5.9	7.4	6.3 (-10.2, -3.9)	5 (31)	11 (69)
	CKD-EPI	0.916***	12.4	−4.8	5.9	6.7 (-8.7, -1.9)	5 (31)	11 (69)
	CG-CrCl	0.893***	14.4	−1.1	4.0	7.6 (-5.6, 1.9)	8 (50)	13 (81)
CrCl > 60 (*N* = 11)	mCrCl	0.677*	10.3	0.7	11	17.8 (-11.5, 6.3)	10 (91)	11 (100)
	4vMDRD	0.214º	15.5	−22.0	24.4	22.1 (-34.5, -12.4)	2 (18)	8 (73)
	CKD-EPI	0.153º	16.8	−18.4	19.0	23.6 (-25.5, -1.9)	4 (36)	10 (91)
	CG-CrCl	0.247º	20.3	−8.1	9.4	13.0 (-17.8, -4.8)	4 (36)	10 (91)
CrCl 30 to 60 (*N* = 11)	mCrCl	0.756**	7.0	−3.8	5.9	7.8 (-5.2, 2.7)	10 (91)	11 (100)
	4vMDRD	0.744**	4.7	−8.0	8.0	5.4 (-10.8, -5.4)	4 (36)	9 (82)
	CKD-EPI	0.731*	5.0	−7.0	7.0	5.1 (-10.8, -5.7)	3 (27)	9 (82)
	CG-CrCl	0.695*	7.1	−3.3	5.1	6.8 (-7.5, -0.6)	6 (55)	11 (100)
CrCl < 30 (*N* = 9)	mCrCl	0.767**	3.9	1.4	1.5	2.3 (-0.2, 2.1)	8 (89)	8 (89)
	4vMDRD	0.672*	2.8	−6.2	6.2	3.0 (-7.2, -4.2)	2 (22)	7 (78)
	CKD-EPI	0.593º	3.4	−6.4	6.4	4.0 (-8.2, -4.2)	2 (22)	7 (78)
	CG-CrCl	0.300º	7.4	−0.6	4.2	6.0 (-4.2, 1.9)	4 (44)	7 (78)
BMI > 30 (*N* = 8)	mCrCl	0.989***	6.8	0.1	2.7	7.3 (-5.9, 1.4)	7 (88)	8 (100)
	4vMDRD	0.876**	21.2	−10.3	16.3	19.7 (-25.5, -5.9)	0	3 (38)
	CKD-EPI	0.861**	24.6	−8.8	14.1	16.2 (-22.0, -5.9)	0	6 (75)
	CG-CrCl	0.874**	25.5	−6.1	7.1	9.5 (-7.7, 1.8)	4 (93)	7 (88)
BMI > 25 bis 29.9 (*N* = 16)	mCrCl	0.956***	10.8	−0.4	4.3	8.1 (-4.3, 3.8)	14 (88)	15 (94)
	4vMDRD	0.963***	7.7	−6.8	6.8	10.3 (-14.2, -3.9)	7 (44)	13 (81)
	CKD-EPI	0.972***	7.5	−6.7	6.7	9.0 (-12.0, -3.0)	5 (31)	13 (81)
	CG-CrCl	0.963***	8.0	−3.1	7.8	10.7 (-10.0, 0.7)	9 (56)	14 (88)
BMI < 24.9 (*N* = 7)	mCrCl	0.984***	6.5	0.8	5.9	(-11, 0.8)^††^	7 (100)	7 (100)
	4vMDRD	0.983***	5.6	−11.0	11.0	(-33.0, -5.3)^††^	1 (14)	6 (86)
	CKD-EPI	0.971***	8.7	−6.0	6.0	(-26.0, -0.8)^††^	3 (43)	7 (100)
	CG-CrCl	0.950***	10.7	−4.2	4.2	(-32.7, 1.8)^††^	4 (57)	7 (100)

***p < 0.001; **p < 0.01; *p < 0.05; ºnon significant

^†^displayed in mL/min/1.73 m^2^; ^††^(Minimum, Maximum)

^a^ *p* < 0.005 vs the three other methods; ^b^ *p* = 0.07 vs 4vMDRD; ^c^ *p* = 0.21 vs CKD-EPI;^d^ *p* = 0.50 vs CG

In the case of one specific female patient suffering from chronic autoimmune disease, medically examined for proteinuric nephropathy, the BCM based model performed overwhelmingly better than the comparator prediction methods (see Fig. 1). Unlike the other subjects in the study, this patient was rather young (21 years), had a high BMI (35.1 kg/m^2^), and normal measured CrCl of 83.5 mL/min/1.73 m^2^ with a plasma creatinine in the “low normal range”. Regarding her BIA values, we observed a rather low ratio of BCM to total body weight (0.25). Excluding this “outlier” case from the analyses resulted in a convergence of the correlation parameters R and SEE, without substantially affecting both accuracy and precision analyses substantially (see Table 6). This finding applied for the unstratified validation group as well as the respective BMI (>30), and CrCl strata (24hU-CrCl > 60 ml/min/1.73 m^2^).

**Table 6:** Validation group: Correlations, bias, precision and accuracy of the different GFR prediction methods and measured 24hU-CrCl without statistical single data point outlier

		Correlation coefficient R	SEE^†^	BIAS median difference^†^	ABSOLUTE BIAS^†^	PRECISION IQR (P25, P75) of differences^†^	ACCURACY	
							P15 (%)	P30 (%)
All subjects (*N* = 30)	mCrCl	0.972***	9.0	0.1	4.3	6.9 (-4.4, 2.5)	27 (90)^a^	30 (100)^bcd^
	4vMDRD	0.974***	8.7	−8.6	8.6	14.4 (-19.7, -5.3)	8 (27)	17 (57)
	CKD-EPI	0.974***	8.7	−7.1	7.1	12.9 (-17.1, -4.2)	9 (30)	17 (57)
	CG-CrCl	0.971***	9.3	−4.8	6.6	9.1 (-9.0, 0.1)	17 (55)	28 (93)
Female (*N* = 15)	mCrCl	0.952***	5.6	−0.6	1.8	6.3 (-4.2, 2.1)	14 (93)	14 (93)
	4vMDRD	0.951***	4.8	−6.4	6.4	6.1 (-10.3, -4.2)	5 (33)	11 (73)
	CKD-EPI	0.929***	6.7	−5.3	5.3	6.2 (-8.8, -2.7)	5 (33)	11 (73)
	CG-CrCl	0.952***	6.7	−1.6	2.8	8.0 (-6.2, 1.8)	8 (53)	13 (87)
CrCl > 60 (*N* = 10)	mCrCl	0.638*	14.0	−0.4	11.5	15.3 (-11.7, 3.5)	9 (90)	10 (100)
	4vMDRD	0.668*	13.6	−23.2	23.2	21.4 (-35.3, -13.9)	2 (20)	8 (80)
	CKD-EPI	0.660*	13.7	−18.7	18.7	22.3 (-25.8, -3.5)	4 (40)	10 (100)
	CG-CrCl	0.771**	11.6	−8.7	8.7	14.9 (-20.6, -5.7)	4 (40)	10 (100)
BMI > 30 (*N* = 7)	mCrCl	0.995***	5.2	−0.4	1.5	(-15.5, 1.5)^††^	7 (100)	7 (100)
	4vMDRD	0.991***	7.4	−10.5	10.5	(-42.0, -4.2)^††^	0	3 (43)
	CKD-EPI	0.995***	5.4	−9.1	9.1	(-34.0, -4.2)^††^	0	6 (86)
	CG-CrCl	0.987***	8.9	−7.1	7.1	(-26.6, 6.1)^††^	4 (57)	7 (100)

***p < 0.001; **p < 0.01; *p < 0.05;

^†^displayed in mL/min/1.73 m^2^; ^††^(Minimum, Maximum)

^a^ *p* < 0.005 vs the three other methods; ^b^ *p* = 0.12 vs 4vMDRD; ^c^ *p* = 0.34 vs CKD-EPI; ^d^ *p* = 0.8 vs CG

**Fig. 3:**
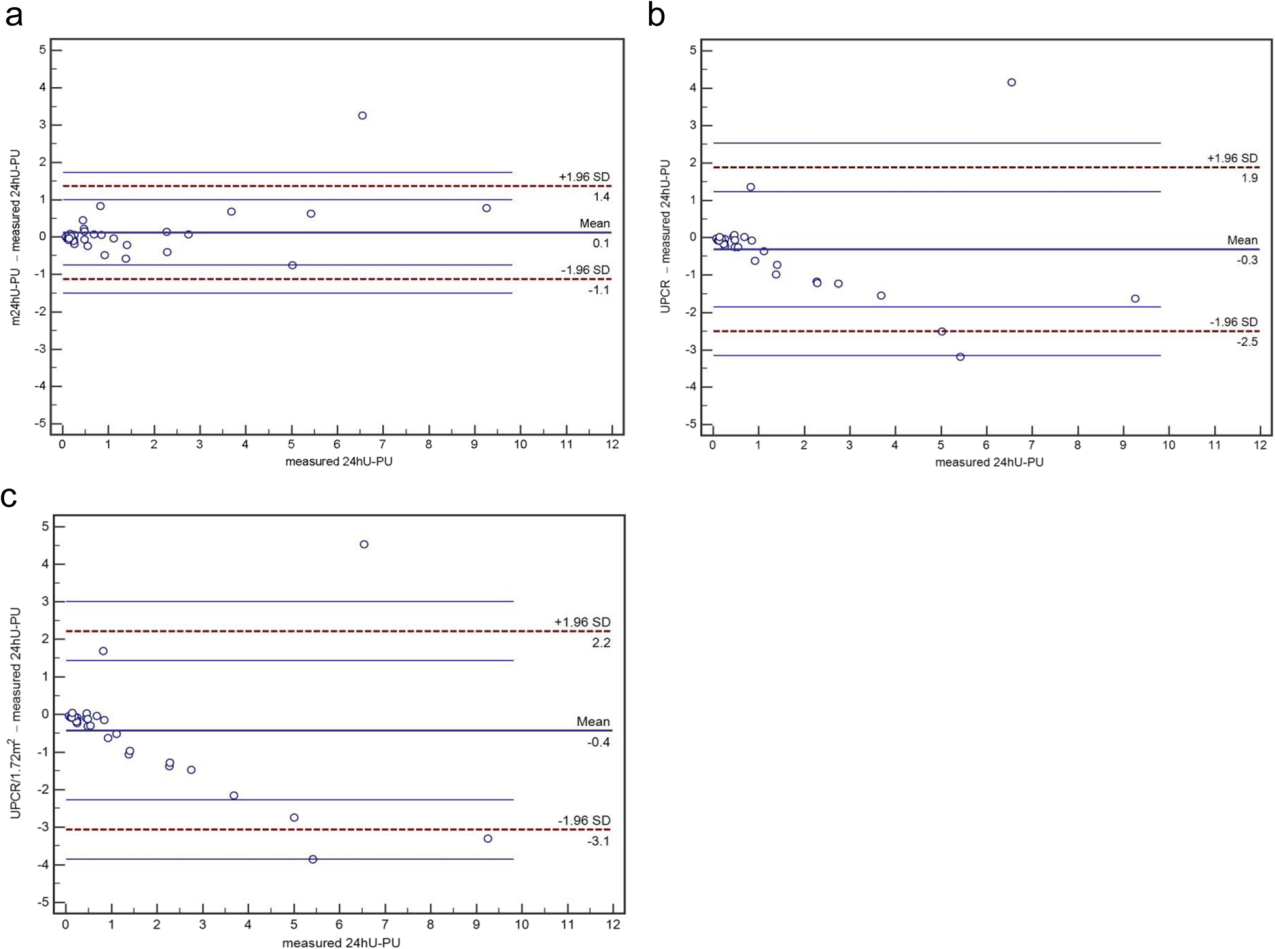
Bland-Altman-Plots of 24 h urinary protein excretion prediction in comparison with measured 24 h urinary protein excretion (24 h-PU) (units of x- and y-axis g/24 h). **a** estimated 24 h urinary protein excretion by m24hU-Cr base: Bias (mean difference) = 0.1 g/24 h, LoA = -1.1, 1.4 g/24 h (dashed lines), 95 % confidence interval of upper and lower LoA (thin lines). **b** estimated 24 h urinary protein excretion by UPCR: Bias = -0.3 g/24 h, LoA = -2.5, 1.9 g/24 h. **c** estimated 24 h urinary protein excretion by UPCR/1.73 m^2^: Bias = -0.4 g/24 h, LoA = -3.1, 2.2 g/24 h

Calculated 24 h urinary protein excretion (*N* = 36) derived from spot UPCR and modified by BCM based 24 h-creatininuria (BCM based pPU) showed a high correlation with measured amount of protein in 24-h urine (*R* = 0.976, *p* < 0.001, SEE = 0.55 g/24 h). In contrast, correlations of UPCR or UPCR normalized for BSA, respectively, with measured 24 h urinary protein excretion, were lower (*R* = 0.791, *p* < 0.001, SEE = 1.28 g/24 h; *R* = 0.862, *p* < 0.001, SEE = 1.10 g/24 h, respectively). The median difference between measured and calculated amount of 24 h urinary protein was -0.02 (IQR = 0.20) g/24 h for the BCM based 24 h-creatininuria method. In contrast, calculated protein excretion from UPCR, normalized or not normalized for BSA, differed from the total of measured protein in 24-h urine by median 0.08 g/24 h (IQR = 0.12) and 0.27 g/24 h (IQR = ±0.63), respectively. Accuracy, analysed as percentage of estimates lying within 15 % (P_15_) or 30 % (P_30_), showed a superior performance of the BCM based pPU (P_15_ = 50 %; P_30_ = 83 %) over protein excretion calculated from UPCR normalized (P_15_ = 8 %; P_30_ = 22 %) and not normalized (P_15_ = 17 %; P_30_ = 61 %) for BSA (*p* < 0.05, *p* < 0.001, respectively). Bias and agreement of the different methods to estimate urinary protein excretion are shown in Fig. 3. The most striking difference regarding accuracy, however, could be observed in a subgroup of 11 individuals with measured 24hU proteinuria above 1000 mg. Whereas in BCM based pPU P_15_ showed a high degree of accuracy of 73 % (P_30_ = 100 %), P_15_ for UPCR, normalized or not normalized for BSA, indicated lesser accuracy estimates, with P_15_ of 0 for both, and P_30_ of 0 and 18 %, respectively.

## Discussion

In the present study, we demonstrate and confirm that a model using body cell mass (BCM) determined by tetrapolar single frequency bioimpedance analysis (BIA) provides accurate estimations of creatinine clearance over a wide range of renal function, both in patients with nephropathy and in healthy individuals. This BCM based model proved to be superior to estimation formulas such as the Cockcroft-Gault, 4vMDRD or CKD-EPI formulas that are solely derived from plasma creatinine measurements. Moreover, this approach is clearly less cumbersome than determination of the clinical gold standard of creatinine clearance derived from 24 h urine collection. In addition to previous studies using a similar concept, we have extended the model to predict 24-h protein excretion from a single spot urine [22, 23].

Estimation of excretory renal function in clinical practice is usually based on estimation methods derived from plasma creatinine measurements. The latter – and thereby the accuracy of these methods regarding GFR determination – is affected not only by its renal elimination, but also its production, which depends on several factors. Among those, the far most important are muscle mass, and, to a much lesser extent, nutritional aspects. Finally, age, gender, ethnicity and genetic variability may affect creatinine production unrelated to muscle mass.

BIA technology has been shown previously to provide accurate determination of body composition in various stages of CKD, including patients undergoing maintenance hemodialysis [30–33]. As demonstrated by Donadio et al., body cell mass determined by tetrapolar single frequency BIA correlated best with urinary creatinine excretion [21–23]. BCM, extracellular (ECM) and fat mass (FM) constitute the three body compartments as described in detail elsewhere [27]. Lean body mass (LBM), which we obtained from the skinfold measurements method, can be used synonymously with FFM for our purposes. BCM in contrast is defined as the total mass of all cells in the body that constitutes all metabolically active tissues and, therefore, unlike FFM or LBM, excludes extracellular mass (ECM). Hence, it most accurately reflects the total of all muscle fiber containing cells, including both skeletal and smooth muscle cells, thereby explaining the good correlation with excreted creatinine in our study as well as in those mentioned above. In contrast, other anthropometric parameters like waist/hip ratio or skin fold based measurements did not contribute substantially to the model predicting CrCl. Likewise, tetrapolar BIA performed substantially better compared to the handheld BIA device used for comparison. Like others, we developed and validated our model by using training and a validation group [22, 23]. In contrast to previous studies, however, additional variables were measured and tested to be incorporated into our prediction formula, resulting in a regression model containing BCM and gender.

Unstratified, the BIA derived method yielded the best results of concordance and accuracy with the measured CrCl among all tested models. Stratified for body weight, the BIA based prediction formula seemed to perform best in subjects with BMI > 30 kg/m^2^. Likewise, the newly developed prediction method excelled in the subgroup of patients with measured CrCl > 60 mL/min/1.73 m^2^. However, even in lower range strata of CrCl or BMI, the model was overall superior to all other estimation methods. With regard to the statistical outlier patient with characteristics reflecting ‘sarcopenic obesity’, it seems that the BIA based GFR estimation method outperforms the other equations most obviously. The substantial impact of inclusion versus exclusion of this outlier patient on correlation coefficients and SEE has to be interpreted in context of the small subgroups.

Although a correct 24 h urine collection remains the gold standard to quantify proteinuria, urinary protein/creatinine ratio is broadly accepted (UPCR) for assessing and monitoring the course of proteinuric nephropathy. With the denominator of UPCR being expressed in grams of creatinine, the nominator is usually considered to reflect the amount of 24 h urinary protein excretion, as the average individual 24-h creatinine excretion for the population is approximately 1000 mg/day per 1.73 m^2^. Considering the fact that some individuals’ creatinine excretion per day will markedly differ from 1 g (e.g. muscle mass differing from average or due to changing body composition), it appears worthwhile to establish a better method to more accurately estimate proteinuria. Towards this aim, using UPCR from a spot urine sample extrapolated to estimated 24 h urinary protein excretion based on 24 h-creatininuria derived from BIA measurement, significantly improves the quantification of 24 h urinary protein excretion. However, this approach, too, does not account for circadian variability in urinary protein excretion.

Several limitations apply to our findings. First, all methods examined were compared to measured CrCl rather than GFR. Thus, for the 4vMDRD and CKD-EPI formulas, which refer to GFR, systematic underestimation to measured CrCl results, which partly explains the lower accuracy and larger bias of these estimates. Nevertheless, comparison between the BCM based method and the common estimation formulas referring to measured CrCl not only revealed no systematic difference in the former in contrast to the latter, but also less variation. Second, it could be argued that methods to predict excretory kidney function should be evaluated against the gold standard for measured GFR, such as radionuclide techniques. However, we deliberately chose CrCl from timed urine collection instead, as this represents the universal clinical standard for routine determination of measured excretory renal function [34, 35]. Moreover, pharmaceutical dosing information is traditionally given for CrCl strata and in reference to the Cockcroft-Gault formula for its estimation [36]. Third, a new GFR prediction method based on serum cystatin C has recently been published [37]. In our study, we did not examine cystatin C, therefore, it could not be compared to our model. Cystatin C, which is less dependent on muscle mass than creatinine, may be a useful marker for kidney function in subjects with body composition outside of the normal range, too. Fourth, all methods to determine renal function based on creatinine, including ours, are limited by its tubular secretion, which is proportionally higher in the lower range of excretory kidney function (e.g. GFR below 20 mL/min/1.73 m^2^). However, this limitation is usually outweighed in subjects with abnormal body composition. Furthermore, it has to be emphasized that the investigated population exclusively consisted of Caucasians, formally limiting the extrapolation of our findings to individuals of other ethnicity. Finally, the BIA technology applied in our study used single frequency rather than multifrequency measurements, which, theoretically, could have resulted in even better prediction of body compartments, and, thus, estimated CrCl.

## Conclusion

In summary and conclusion, the method presented herein to predict creatinine clearance and urinary protein excretion based on a model using estimated urinary creatinine excretion determined by measurement of body cell mass by bioimpedance (BIA) technique has proven to be both accurate and convenient to quantify renal function in normal and diseased states. This method may become particularly helpful for the evaluation of patients with borderline renal insufficiency and/or with abnormal body composition as well as in ethnical groups other than those used for development and validation of the established estimation formulas.

## Abbreviations

GFR: Glomerular filtration rate
eGFR: Estimated GFR
CrCl: Creatinine clearance
24hU-CrCl: Measured CrCl (based on a 24-hour urine collection)
PCr: Plasma creatinine
CKD-EPI: Chronic kidney disease epidemiology collaboration
4vMDRD: 4 variables modification of diet in renal disease
CG: Cockcroft-Gault formula
CG-CrCl: Creatinine clearance estimation by Cockcroft-Gault formula
24hU-Cr: 24 h creatininuria
m24hU-Cr: Model based 24 h creatininuria
mCrCl: model based creatinine clearance
UPCR: Urinary protein creatinine ratio
24h-PU: 24 h urinary protein excretion (24 h proteinuria)
pPU: Predicted 24 h urinary protein excretion resp. proteinuria
BMI: Body mass index
BSA: Body surface area
BIA: Bioimpedance analysis
LBM: Lean body mass
FFM: Fat-free mass
BCM: Body cell mass
ECM: Extracellular mass
TSF: Triceps skin fold
MAC: Midarm circumference
MAMC: Midarm muscle circumference
DXA: Dual x-ray absorptiometry
SEE: Standard error of estimate
R: Correlation coefficient
SD: Standard deviation
IQR: Interquartile range
P25: P75, 25^th^, 75^th^ percentile (quartiles)
P_15_: Range of 15 % difference
P_30_: Range of 30 % difference
LoA: Limits of agreement (mean difference ± 1.96 of SD)
